# Two-State Stochastic Model of In Vivo Observations of Transcriptional Bursts

**DOI:** 10.1007/s13538-025-01785-y

**Published:** 2025-05-08

**Authors:** Luiz Guilherme S. da Silva, Romain Yvinec, Guilherme Prata, Varshant Dhar, John Reinitz, Alexandre F. Ramos

**Affiliations:** 1 Escola de Artes, Ciências e Humanidades, Universidade de São Paulo, Av. Arlindo Béttio, 1000, São Paulo, 03828-000 SP Brazil; 2 Centro Universitário Lusiada - UNILUS, Santos, 11015-101 SP Brazil; 3 PRC, INRAE, CNRS, Université de Tours, Nouzilly, 37380 France; 4 Université Paris-Saclay, Inria, Inria Saclay, Palaiseau, 91120 France; 5 Instituto Federal de Educação, Ciência e Tecnologia de São Paulo, Catanduva, 01109-010 SP Brazil; 6 Department of Statistics, Ecology and Evolution, Molecular Genetics and Cell Biology, University of Chicago, 5734 S. University Avenue, Chicago, 60637 IL USA; 7 Ripple, New York City, 10011 NY USA; 8 Instituto do Cancer do Estado de Sao Paulo, Icesp, Hospital das Clinicas, Faculdade de Medicina, Universidade de São Paulo, São Paulo, 01246-903 SP Brazil

**Keywords:** Gene regulation, Transcriptional bursting, Stochastic modeling

## Abstract

In vivo measurements of gene expression in single cells show behavior that has been interpreted as stochastic bursts of transcription. In one case, these data have been interpreted as random ON–OFF transitions of the gene, but there is no experimental measurements or theoretical treatment of the number of transcripts produced at each burst event. In another case, such data have been interpreted to indicate multiple underlying transcriptional states. Here, we place both of these experiments in a common theoretical framework. In it, we couple two stochastic processes, one for synthesis of transcripts and one for their removal. Analysis of the resulting model is greatly aided by the existence of exact solutions of the master equation. We find the bursting limit of the exact solutions for our two-state gene expression model and show the occurrence of bursts of multiple sizes and durations by exact stochastic simulations. We also demonstrate that data from *Drosophila melanogaster* interpreted in terms of multiple underlying transcription states is fully compatible with underlying two-state ON or OFF transcriptional behavior. We discuss what experimental data is required to unambiguously determine the number of underlying promoter states.

## Introduction

The control of transcription in cells is characterized by intrinsic fluctuations which result from the small number of molecules orchestrating this process. High resolution in vivo experiments in eukaryotes reveal intrinsic fluctuations in the form of bursts of transcription of variable size and duration [1–13]. The existence of these fluctuations was established either with luciferease assays at the protein level or, at the RNA level, by reporters that have repeated binding sites for specific RNA binding proteins which have been fused with flourophores. Of these RNA assays, a particularly important class involves the visualization of nascent elongating transcripts. The quantitative characterization of the stochastic behavior revealed by these bursts is an important scientific question. Such characterization requires the inference of key biophysical parameters from the bursts in terms of some underlying model.

These models all contain some form of the so-called “random telegraph” equation [14–22] in which the switching on and off of transcription is characterized by a pair of first order rate constants for the ON to OFF and OFF to ON transitions. The random telegraph equation has a well-known exact solution in terms of exponentials. Other reaction steps leading to the observed signal are also stochastic, including but not limited to elongation itself, the release of a nascent transcript by the polymerase, RNA processing, and translation. Models exist for these processes, but the corresponding master equations do not have exact solutions [16]. Moreover, many involve unobservable state information such as the exact elongation state of an individual RNA polymerase. In the case of protein reporters, assuming the independence of transcription and translation permits a separation of random process into independent master equations, each of which has an exact solution. In the case of nascent transcripts, all steps subsequent to those described by the random telegraph equation are described deterministically in the interpretation of experiments [5, 6, 10, 20].

**Fig. 1:**
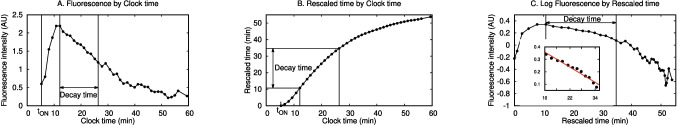
Exponential decay of burst in the rescaled time. **A** The fluorescence intensity of a single burst, using published data [5, Fig. 3C]. The vertical axis shows fluorescence intensity in units of $10^{4}$ larger than the original data. The horizontal shows the clock time in minutes from the end of the 13th nuclear division. The vertical line labeled $t_\textrm{ON}$ indicates the time when the burst starts. The interval denoted by “Decay time” is the period in which the promoter is OFF but signal from elongating transcripts remains detectable. **B** The rescaled time as a function of clock time. **C** The log of fluorescence as a function of the rescaled time. Decay time is shown in rescaled time. The inset shows the logarithm of fluorescence decay while the promoter is OFF

In this paper, we present a phenomenological model of transcriptional fluctuations whose state variables and parameters are very well matched to the experimental observables of in vivo studies of fluctuations in transcription. Like the random telegraph equation, the model presented here has exact solutions and incorporates the ON to OFF and OFF to ON transitions of the bursts. Unlike the random telegraph equation, it also incorporates fluctuations in the loading rate of RNA polymerase and the rate at which transcripts are released by the polymerase. Each of these processes contributes directly to the observed signal in in vivo assays.

Below, we describe the equations and their solutions in detail and demonstrate that they can be applied either to assays of elongating transcripts or to protein reporters. The former is done interpreting experimental data in [5] in terms of a time rescaling proposed in [23]. In the former case, we show that our ON/OFF model produces behavior that was originally thought to require multiple states, and in the latter case, we show that the findings of a previous experimental study can be used in conjunction with our model equation to generate a previously proposed stochastic model of translation. Statistical inference with the model is dependent of detailed experimental circumstances and will be described in a future work.

## Results

Here, we use a two-state master equation model of transcription which has exact solutions given by confluent hypergeometric functions [15, 24] which has been previously applied to the control of the transcription of *even-skipped* stripe 2 in *D. melanogaster* [25]. The model has two stochastic variables. One is the state of the gene’s promoter, which can be ON or OFF. The second stochastic variable, denoted by *n*, indicates the number of molecules of RNA and assumes non-negative integer values. The probabilities of finding *n* molecules in the ON or OFF state are given respectively by $\alpha _n(t)$ and $\beta _n(t)$. In this work, we consider the steady-state limit when probabilities are constant in time and write $\alpha _{n}(t)\equiv \alpha _n$ and $\beta _{n}(t) \equiv \beta _n$. The master equation is given by

1 $$\begin{aligned} 0\!=\! &   k(\alpha _{n-1}-\alpha _{n}) \!+\! \rho [(n\!+\!1)\alpha _{n+1}\!-\!n\alpha _{n}] \!-\! h\alpha _{n} \!+\! f\beta _{n}, \\ 0= &   \rho [(n+1)\beta _{n+1} - n\beta _{n}] + h\alpha _{n} - f\beta _{n}, \end{aligned}$$

where *k* is the synthesis rate, *f* and *h* are the rates of OFF to ON and ON to OFF transitions respectively, and $\rho$ is the degradation rate.

**Fig. 2:**
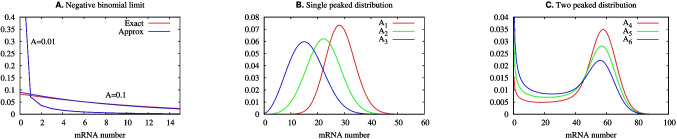
Here, we show the probability distributions obtained with (3) and (5). The Fano factors for those distributions are given by $1+N(1-A)/(1+\epsilon )$. **A** The approximation of (3) by (5) with parameters $(N,\epsilon )= (100,10)$ and the value of *A* is given as a key within the graphs. The Fano factors for $A=0.01$ and $A=0.001$ are, respectively, 10. and 10.08. Where the red trace is not visible, the curves overlap. **B** Single peaked probability distributions of exact solutions of (3) for the parameters $(N,\epsilon )=(28.9,7.2)$ and $(A_1,A_2,A_3)=(0.99,0.77,0.55)$ with the respective Fano factors given by $\approx (1.04, 1.81, 2.59)$. **C** Two-peaked probability distributions of the exact solutions of Eq. (3) for the parameters $(N,\epsilon )=(60,0.75)$ and $(A_4,A_5,A_6)=(0.8,0.7,0.6)$ with the respective Fano factors given by $\approx (7.86, 11.29, 14.71)$. Note that one may compute the probabilities given by (3) without an explicit value of $\rho$

For modeling Suter and collaborator’s data, we interpret *n* as the number of mature mRNA molecules, and hence, $\rho$ is just the degradation of mRNA’s as usual. We change the interpretation of the model when analyzing the study by Bothma and coworkers since they indirectly observe the number of nascent, elongating, mRNA molecules which will be our *n*. The binding of PolII to the promoter site when it is ON, and the reading of the DNA to transcribe it until releasing of the transcript, is approximated as a birth and death process. The former is the PolII binding—and—initiation of RNA synthesis while the completion of transcription is the latter. Hence, the degradation of an elongating RNA is the conclusion of a complex process of PolII traffic through the DNA [16]. However, that may still be modeled as a Poissonian birth and death process under quite general circumstances [23] as we show in the analysis presented in Fig. 1. Here on, we refer to *n* as the number of mRNA molecules, and its interpretation will be straightforward according to context.

In [23], a complex birth and degradation process is modeled as Poissonian by means of a rescaling of clock time in terms of an integral of the survival density. In the experiments, the survival density relates to the fluorescence intensity decay during observation of transcription elongation. In Fig. 1A, we show how the fluorescence intensity decays in [5] and highlight the range during which the promoter is OFF. Then, in Fig. 1B, we show the result of the temporal rescaling by numerically computing the integral of the fluorescence in time. The logarithm of the fluorescence intensity plotted as function of the rescaled time is shown in Fig. 1C, and the inset shows the linear decay of fluorescence within the time interval during which the promoter is OFF. That demonstrates that there is an exponencial decay as expected for a Poissonian birth and death process and suggests the application of our model for phenomenologically interpreting the experimental data presented by Bothma and coworkers.

The steady-state probabilities of finding *n* mRNA molecules independently of the gene state are denoted by $\phi _n$, with $\phi _n = \alpha _n+\beta _n$. This quantity has been shown [26] to be given by the equation

2 $$\begin{aligned} \phi _n = \frac{N^n}{n!} \frac{(A \epsilon )_n}{(\epsilon )_n} \textrm{M}(A \epsilon + n,\epsilon + n,-N), \end{aligned}$$

where $\textrm{M}(a,b,z)$ denotes the KummerM function, and $(x)_n=x(x+1)\dots (x+n-1)$ denotes the Pochhammer function with $(x)_0=1$. Equation (3) is written in terms of the parameters $(N,\epsilon ,A)$ defined by

3 $$\begin{aligned} N=\frac{k}{\rho }, \ \ A = \frac{f}{f+h}, \ \ \epsilon = \frac{f+h}{\rho }. \end{aligned}$$

*N* denotes the expectation of the number of mRNA molecules when the promoter is exclusively ON. The steady-state probability of finding the promoter in the ON state is denoted by *A*, with $A=\sum _{n=0}^\infty \alpha _n$. $\epsilon$ gives the ratio of the switching rate between ON and OFF to the rate of mRNA degradation. Thus, $\epsilon \gg 1$ implies that the gene switches multiple times during the mean lifetime of an mRNA molecule, while $\epsilon \ll 1$ implies that the gene stays ON or OFF for a time that is longer than the lifetime of the message. Of the two experimental studies mentioned above, one [3] reports data for which $A \ll 1$, while the other reports data where $\epsilon \gg 1$. We consider each case in turn below.

**Fig. 3:**
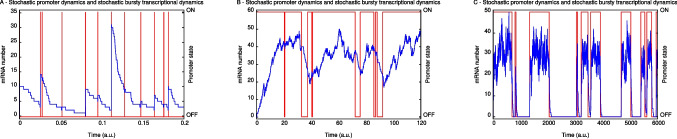
Realizations of the dynamics of the number of mRNA’s and corresponding promoter state by the exact simulation algorithm of Gillespie [28] are presented. The left and right side vertical axes show the number of mRNA molecules and promoter state, respectively. The number of mRNA molecules (promoter state) trajectory is indicated in blue (red). **A**, **B**, and **C** were constructed using parameters $(N, \epsilon , A, \rho )$, respectively, equal to $(8\times 10^3, 10^3, 10^{-3}, 1)$, (40, 5, 0.9, 0.002), and $(30, 0.1, 0.5, 10^{-3})$

**Fig. 4:**
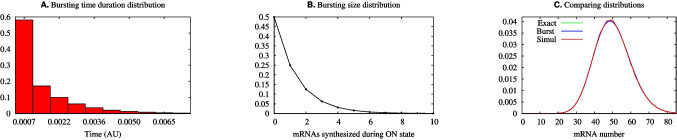
The histograms for the burst duration and burst size for parameters $(N,\epsilon ,A)=(10^4,10^4,0.005)$ are presented in **A** and **B**, respectively. **C** shows a comparison of the steady state distribution of the mRNA numbers obtained from (3), denoted by the label “Exact,” (5), denoted by “Burst,” with the simulations of the trajectories, denoted by “Simul” on the key. The Fano factor here is 1.99

### The Case of $A\ll 1$

We provide a biological interpretation of the bursting limit presented in Eq. (5) in terms of the parameter relations that were used. The choice of $A \ll 1$ implies that the ON$\rightarrow$OFF rate $h \gg f$, the OFF$\rightarrow$ON rate. Together with the limit $\epsilon ,N \gg 1$, these relationships mean that *k* and *h* are the dominant reaction rates in the bursting limit, and, furthermore, that $h \gg \rho$. Biologically, this means that the promoter ON time is shorter than the mean lifetime of the mRNA’s. In other words, because $h\gg \rho$, most of the mRNA degradation occurs while the promoter is OFF. Also, because $h\gg f$, the promoter tends to be ON for short periods separated by long intervals of OFF. For the short time interval when the promoter is ON, because of the high value of *k*, a large number of mRNA molecules are synthesized. Because mRNA synthesis and ON-OFF switching of the promoter are both stochastic processes, the number of RNA molecules produced in a burst and its time duration are both random variables and will have different values at each bursting event.

Because *A* is a measure of the proportion of time that the gene is ON, setting *A* very small in the bursting limit is a mathematical expression of the commonsense point that to observe individual bursts, they should be well separated from one another in time. This is apparent in Fig. 2A, where it is evident that $\sum _{n=0}^\infty | \phi _n - \tilde{\phi }_n | \rightarrow 0$ as $A\rightarrow 0$. The trajectories of Fig. 3A further illustrate this fact. The number of mRNA molecules (Fig. 3A) increases rapidly during the very short time interval when the promoter is in the ON state, and then decays exponentially after the promoter switches to the OFF state. Figure 4A and B are histograms for, respectively, the burst duration and size. Both are random variables arising from intrinsic fluctuations within the cell.

### The Case of $\epsilon \gg 1$

Figure 2B shows unimodal probability distributions for high values of $\epsilon$ and a range of values of *A*. The distribution of the number of mRNA molecules in this figure are well approximated by a Poisson distribution when *A* is closer to 1. In Fig. 2B, as $A\rightarrow 1$, the mode of the distribution moves to the right and its profile approaches that of a Gaussian distribution. The corresponding trajectories of the promoter state and of mRNA numbers are shown on Fig. 3B. The number of mRNA molecules fluctuates around its stationary mean value. The ON and OFF promoter states tend to have durations proportional to *A* along a sufficiently long time interval. The bursts still occur when the promoter is ON, but, because of the slow mRNA synthesis rate in comparison with the duration of the ON states, the trajectories of mRNA numbers in Fig. 3B have less abrupt increases than Fig. 3A.

Data very similar in appearance to Fig. 3B appears in Fig. 4A of a study of transcription driven by the *even-skipped* stripe 2 enhancer in single nuclei of live embryos of *D. melanogaster* [6]. The peaks of differing height were interpreted as individual bursts revealing multiple ON promoter states of differing synthetic capacity through the use of a deterministic model of PolII binding and elongation. The peaks of multiple heights and duration seen in Fig. 3B show that the time courses of transcript number observed by Bothma and collaborators [6] can be obtained from two promoter states only. The fact that we are considering the number of elongating transcripts, and Bothma and collaborators report data about the fluorescence intensity is of low impact since there is a direct relation between the two quantities, as presented previously [5].

### Bursting Limit

In the work of Suter et al.[3], Fig. 2A shows that the number of mRNA molecules varies from 0 to 8 and is frequently 0. This indicates that $A \ll 1$. We now show that in this limit with the conditions $\epsilon ,N \gg 1$, and the ratio $\delta = N/\epsilon$ set to a finite constant, the steady-state solutions in (3) for the master equations (1) and (2) have the negative binomial probability distribution as a particular case. That transforms $\phi _n$ into $\tilde{\phi }_n$ where

4 $$\begin{aligned} \phi _n \rightarrow \tilde{\phi }_n = \frac{(A \epsilon )_n}{n!} \left( \frac{\delta }{1+\delta }\right) ^n \left( \frac{1}{1+\delta }\right) ^{A \epsilon }, \end{aligned}$$

is the negative binomial distribution which governs mRNA numbers in transcriptional bursts, as has previously been shown for translational bursts [27].

For simplicity, we demonstrate this in the framework of the generating functions of the probabilities $\phi _n$. In that formalism, we consider the probabilities as coefficients of a series expansion of an analytic function, namely $\phi (z) = \sum _{n=0}^\infty \phi _n z^n$. This technique has been used to obtain the closed form for $\phi _n$, and its generating function is $\phi (z) = \textrm{M} (A\epsilon , \epsilon , N(z-1))$. Now, we obtain the generating function of the probabilities $\tilde{\phi }_n$ by recalling a Laplace transform of a hypergeometric function:

$$\begin{aligned}&\textrm{M}(A\epsilon ,\epsilon ,N(z-1)) = \frac{1}{\Gamma (A\epsilon )}\\&\int _0^{\infty } \textrm{e}^{-t} t^{A\epsilon -1} \textrm{M}(0,\epsilon ,N(z-1)t)dt, \end{aligned}$$

introducing the rescaling

5 $$\begin{aligned} N = \epsilon \delta , \end{aligned}$$

and noting the fact that, as $\epsilon \rightarrow \infty$,

$$ \textrm{M}(0, \epsilon ,\epsilon z) \rightarrow \textrm{e}^z. $$

By convergence under the integration, we have

$$\begin{aligned} \textrm{M} (A\epsilon , \epsilon , N(z-1))\sim &   \frac{1}{\Gamma (A\epsilon )} \int _0^{\infty } \textrm{e}^{-t} t^{A\epsilon -1} \textrm{e}^{\delta (z-1)t} dt \end{aligned}$$

6 $$\begin{aligned}\sim &   \frac{1}{\Gamma (A\epsilon )} \int _0^{\infty } \textrm{e}^{-(1+\delta (1-z))t} t^{A\epsilon -1} dt. \end{aligned}$$

Making the variable change $u=(1+\delta (1-z))t$ gives

$$ \frac{1}{\Gamma (A\epsilon )} \int _0^{\infty } \textrm{e}^{-(1+\delta (1-z))t} t^{A\epsilon -1} dt = [1+\delta (1-z)]^{-A\epsilon }, $$

so that the generating function $\phi (z)$ for the probabilities $\phi _n$ can be written as

7 $$\begin{aligned} \phi (z) \sim \tilde{\phi }(z)= \Bigg {(}\frac{1}{1+\delta (1-z)}\Bigg {)}^{A\epsilon }. \end{aligned}$$

That is the generating function of the negative binomial distribution that governs mRNA numbers in transcriptional bursts, as has previously been shown for translational bursts. The probabilities $\tilde{\phi _n}$ are obtained by evaluating *n*-th derivative of the generating function, namely, $\tilde{\phi }_n = \frac{1}{n!} \left. \frac{d^n \tilde{\phi }(z)}{dz^n} \right| _{z=0}$. At the bursting limit, one has $\delta = (k/h)(1+f/h)^{-1} \sim k\frac{1}{h}$. Namely, the expected burst size is the product of the synthesis rate to 1/*h*, which is proportional to the average duration of the ON state.

### Probability Distributions

Figure 2A shows a comparison between the probabilities $\phi _n$ and their approximations $\tilde{\phi }_n$ for two sets of parameters $(N,\epsilon ,A)$, which we denote below as $P_1\equiv (100,10,0.1)$ and $P_2\equiv (100,10,0.01)$. The accumulated difference between the two probability distributions, $\sum _{n=0}^\infty | \phi _n - \tilde{\phi }_n |$, is $< 10^{-1}$ and $< 10^{-2}$, respectively, for the parameter sets $P_1$ and $P_2$. Note that the value of *A* indicates the order of magnitude of the accumulated error of the negative binomial approximation. Figure 2B and C show single peaked and two-peaked probability distributions of exact solutions of (3). The two peaked distributions occur when $\epsilon < 1$ with the relative height of the peaks dependent on the value of *A*. The modes of the single peaked distributions approach *N* as *A* approaches 1.

### Exact Simulations

Figure 3 shows three realizations of the stochastic process governed by the probabilities $\alpha _n(t),\beta _n(t)$. The blue (red) lines show the number of mRNAs (promoter state) as a function of time. Figure 3A shows trajectories for transcriptional bursting in which mRNA numbers at steady state are governed by the negative binomial limit of the probability distribution of the (3). Figure 3B presents trajectories obtained from transcriptional dynamics characterized by a single peaked probability distribution, as in Fig. 2B. The bursting of mRNAs occurs during the promoter ON state and the OFF-ON-OFF state switching time duration appears as a single vertical line. The bimodal distribution limit of Fig. 2C has its corresponding trajectories shown in Fig. 3C.

Figure 4 shows histograms obtained from the simulations at the limit of the negative binomial distribution when bursting takes place. Figure 4A shows a histogram for the time spent by the promoter at the ON state. Figure 4B provides a histogram for the number of mRNA molecules synthesized during each time interval when the promoter is ON. The histogram of the number of mRNA’s after the system has achieved steady state is shown in Fig. 4C, which gives a comparison of the probability distributions produced by the exact solution (3), a simulation of it by the Gillespie algorithm ($10^7$ repetitions) [28], and in the bursting limit (5).

## Discussion

### The Case of $\epsilon < 1$ and Multimodality

The two-state stochastic model presented here suggests the necessary experimental design for probing the underlying state structure of promoters in *Drosophila* and other organisms. Consider the binary model presented here at the limit of bimodal distributions of *n* as shown on Fig. 2C. The average time intervals for the promoter to be in the ON ($T_{\text{ ON }} \propto 1/h$) or OFF ($T_{\text{ OFF }} \propto 1/f$) states are similar to each other but longer than the average lifetime of message, $T_D \sim 1/\rho$, *i.e.* $T_\textrm{ON} \sim T_\textrm{OFF} \gg T_D$. Then, on average, most of the mRNA synthesized during the ON state will be degraded before the promoter switches to the OFF state. When the promoter is OFF, the remaining mRNA will be rapidly degraded. Figure 3C illustrates this fact. In that regime, a histogram of the amount of mRNA would be two peaked. The first peak would be near zero, and the second peak would be determined by the transcription rate of the ON state.

The above reasoning implies that in a slow switching regimen, an *M* state promoter would produce an *M*-modal histogram [19]. Indeed, let us consider a promoter operating in states $\textrm{OFF}, \textrm{ON}_1, \dots , \textrm{ON}_\mathrm{M-1}$ which corresponding synthesis rates $k_0=0$, $k_1, \dots , k_\mathrm{M-1}$ satisfy $0< k_1< k_2< \dots < k_\mathrm{M-1}$. Now, we consider a switching regimen which promoter states have average durations satisfying $T_{\textrm{ON}_1} \sim T_{\textrm{ON}_2} \dots \sim T_{\textrm{ON}_\mathrm{M-1}} \sim T_\textrm{OFF} \gg T_D$. During steady state, the number of products will have a trajectory similar to that of Fig. 3C, though fluctuating around each quantity $N_j=k_j/\rho$. The average time $T_i$ during which the trajectory will fluctuate around each $N_j$ is recovered from somewhat cumbersome relations among the transition rates between promoter states [19]. Note that the $N_j$’s must differ by a sufficiently large quantity such that fluctuations around them would not cause an undistinguishable merging of two neighboring peaks. As an example, one might inspect Fig. 2C considering that $N \rightarrow 0$, which would lead the right peak to merge with the left one.

### Applicability of our Description to Experimental Data

The experimental realization of the *M*-state regime depends on the system used[10]. In [6], observations are made of the fluorescence level of spots of nascent, elongating transcripts. As previously mentioned, for these experiments, our synthesis rate $k_i$ represents the average rate of initiation of new transcripts while $\rho$ represents the rate of release of completed transcripts from the DNA rather than their physical destruction. Bothma and coworkers [6] model the elongation of transcripts deterministically by a constant elongation rate. Thus, spots have a three phase fluorescence profile which rises during the RNA polymerase loading phase, is flat when initiation and release of transcripts is in equilibrium, and declines while release takes place in the absence of loading. The authors then ascribe varying peak heights to differing discrete loading rates. In this work, we show that the differing peak heights can be explained by intrinsic fluctuations.

These alternatives can be further distinguished by experiments on a wider variety of constructs. Varying the length of transcripts would, in our model, vary $\rho$ with associated stochastic effects, while in the model of Bothma and coworkers, it would result in an extended plateau phase. Alternatively or in tandem, an enhancer-basal promoter construct could be constructed with slower switching between the ON and OFF states in comparison with the rate of transcript elongation and release from DNA. In such conditions, multiple underlying ON states would be reflected by multi-peaked histograms, with the caveat that any states with extremely fast switching times would be missed. In this approach, one may expect the spot’s fluorescence levels to be governed by a bimodal (or multi-modal) probability distribution when the OFF state of the promoter is longer than the time required for the releasing of the RNA’s. Furthermore, the transcriptional bursting is the release of multiple transcripts during a very short time interval because of multiple RNA Pol II binding to the ON promoter. In this approach, the bursting size might be inferred from the average value of the fluorescence distributions of spots at each bursting event and a full correspondence between the two approaches of Refs. [3, 6] could be established.

An experiment aiming to verify the applicability of the two-state model would require measurements of distributions of the burst size distribution, the ON and OFF states durations, and the mRNA number (or the spot’s fluorescence levels) in the steady-state regime. The parameter $\delta$ would be estimated from the average burst size, and the average durations of the ON ($T_\textrm{ON}$) and OFF ($T_\textrm{OFF}$) states would be used to estimate *A* by means of ${T_\textrm{ON}}/{(T_\textrm{ON}+T_\textrm{OFF})}$. Finally, the average mRNA numbers $\langle n \rangle$ would be useful on estimating *N* by means of $N=\langle n \rangle / A$ while the value of $\epsilon$ would be obtained from $\epsilon = N/\delta$. In the event that the clean experimental regime described above is unobtainable, it is possible that a two-state ON-OFF gene could be distinguished from one with multiple states by carefully comparing the durations of productive and degradative periods. Depending on the resolution of the observations, reasonable statistics on the number of mRNA’s synthesized per production event may also be obtained. In both the two-state and multistate scenarios, the OFF state (where only degradation occurs) should be exponentially distributed. The distribution of the synthesis period will be dependent on the structure of the underlying promoter states. For example, with a state structure of the form OFF, ON$_1$, ON$_2$,..., ON$_M$ where the synthesis rate of $\textrm{ON}_i < \textrm{ON}_{i + 1}$ and only state transitions that increased or lowered *i* by 1 were permitted, the synthesis times would follow a Gamma distribution. On the other hand, if the observations show a geometric burst size distribution, this favors the bursting limit of the two-state model, given by Eq. (5). However, if the bursting limit does not apply, more complex production distribution may occur [29].

The multimodality on the number of products reflects the coupling of multiple stochastic processes governed by single peaked distributions as discussed for two-state stochastic models [30, 31]. For example, each of those may be produced by individual Poissonian-like birth and death processes depending on a parameter $\lambda _i$ that in this case is the ratio of the synthesis to the degradation rate. The parameter of a Poissonian distribution determines the position of its peak. The existence of multiple promoter states imply on the existence of multiple synthesis rates and $\lambda _i$’s. Alternatively, one might have a fixed synthesis rate while the degradation rate would be modulated. That might be the case of random changes on the amounts of microRNAs responsible for the degradation of a given mRNA [32]. One may expect that that would also produce multipeaked distributions, but further theoretical investigations are needed for a precise description of the problem.

## Data Availability

No datasets were generated or analysed during the current study.

## Code Availability

Codes are available under reasonable request.
